# Cardiovascular Magnetic Resonance Before Invasive Coronary Angiography in Suspected Non–ST-Segment Elevation Myocardial Infarction

**DOI:** 10.1016/j.jcmg.2024.05.007

**Published:** 2024-09

**Authors:** Mayooran Shanmuganathan, Chrysovalantou Nikolaidou, Matthew K. Burrage, Alessandra Borlotti, Rafail Kotronias, Roberto Scarsini, Abhirup Banerjee, Dimitrios Terentes-Printzios, Alex Pitcher, Edit Gara, Jeremy Langrish, Andrew Lucking, Robin Choudhury, Giovanni Luigi De Maria, Adrian Banning, Oxford Acute Myocardial Infarction (OxAMI) Study Investigators, Stefan K. Piechnik, Keith M. Channon, Vanessa M. Ferreira

**Affiliations:** a Acute Vascular Imaging Centre, University of Oxford, John Radcliffe Hospital, Oxford, United Kingdom; b Oxford Centre for Clinical Magnetic Resonance Research, John Radcliffe Hospital, National Institute for Health and Care Research Oxford Biomedical Research Centre, Oxford British Heart Foundation Centre of Research Excellence, University of Oxford, Oxford, United Kingdom; c Oxford University Hospitals National Health Service Foundation Trust, John Radcliffe Hospital, Oxford, United Kingdom; d Faculty of Medicine, University of Queensland, Brisbane, Australia; e Institute of Biomedical Engineering, Department of Engineering Science, University of Oxford, Oxford, United Kingdom

**Keywords:** acute coronary syndrome, CMR, early cardiac magnetic resonance, MINOCA, myocarditis, NSTEMI

## Abstract

**Background:**

In suspected non–ST-segment elevation myocardial infarction (NSTEMI), this presumed diagnosis may not hold true in all cases, particularly in patients with nonobstructive coronary arteries (NOCA). Additionally, in multivessel coronary artery disease, the presumed infarct-related artery may be incorrect.

**Objectives:**

This study sought to assess the diagnostic utility of cardiac magnetic resonance (CMR) before invasive coronary angiogram (ICA) in suspected NSTEMI.

**Methods:**

A total of 100 consecutive stable patients with suspected acute NSTEMI (70% male, age 62 ± 11 years) prospectively underwent CMR pre-ICA to assess cardiac function (cine), edema (T_2_-weighted imaging, T_1_ mapping), and necrosis/scar (late gadolinium enhancement). CMR images were interpreted blinded to ICA findings. The clinical care and ICA teams were blinded to CMR findings until post-ICA.

**Results:**

Early CMR (median 33 hours postadmission and 4 hours pre-ICA) confirmed only 52% (52 of 100) of patients had subendocardial infarction, 15% transmural infarction, 18% nonischemic pathologies (myocarditis, takotsubo, and other forms of cardiomyopathies), and 11% normal CMR; 4% were nondiagnostic. Subanalyses according to ICA findings showed that, in patients with obstructive coronary artery disease (73 of 100), CMR confirmed only 84% (61 of 73) had MI, 10% (7 of 73) nonischemic pathologies, and 5% (4 of 73) normal. In patients with NOCA (27 of 100), CMR found MI in only 22% (6 of 27 true MI with NOCA), and reclassified the presumed diagnosis of NSTEMI in 67% (18 of 27: 11 nonischemic pathologies, 7 normal). In patients with CMR-MI and obstructive coronary artery disease (61 of 100), CMR identified a different infarct-related artery in 11% (7 of 61).

**Conclusions:**

In patients presenting with suspected NSTEMI, a CMR-first strategy identified MI in 67%, nonischemic pathologies in 18%, and normal findings in 11%. Accordingly, CMR has the potential to affect at least 50% of all patients by reclassifying their diagnosis or altering their potential management.

Non–ST-segment elevation myocardial infarction (NSTEMI) is highly prevalent and accounts for the majority of all cases of acute MI.^1^, ^2^, ^3^ Typical presentations include chest discomfort with evidence of acute myocardial injury (acute rise and fall in cardiac troponin I [cTnI] concentrations), which may be accompanied by non–ST-segment elevation changes on electrocardiography (ECG). The majority of patients with suspected NSTEMI are referred for invasive coronary angiography (ICA) during their hospitalization, in accordance with standard clinical care pathways.^1^

However, ICA in suspected acute NSTEMI may demonstrate nonobstructed coronary arteries (NOCA) in up to 15% of the patients,^4^ which are often due to nonischemic pathologies (eg, myocarditis or takotsubo cardiomyopathy), or true myocardial infarction with nonobstructed coronary arteries (MINOCA).^5^, ^6^, ^7^, ^8^ Causes of MINOCA include recanalized coronary atherosclerotic plaques, spontaneous coronary artery dissection, coronary thromboembolism, coronary vasospasm, or coronary microvascular dysfunction.^9^^,^^10^ Further, up to 70% of patients presenting with NSTEMI have multivessel disease (MVD) of the coronary arteries on ICA,^11^ which can make identification of the infarct-related artery (IRA) challenging, particularly when ECG findings are ambiguous. Inaccurate diagnosis of an MI (vs nonischemic causes) and incorrect determination of the IRA in MI are frequent clinical challenges in NSTEMI and may lead to suboptimal clinical care and outcomes.

Cardiac magnetic resonance (CMR) is the imaging gold standard for cardiac structure, function, and myocardial tissue characterization;^12^ CMR can distinguish MI from nonischemic causes of acute myocardial injury, and accurately determine the territory of acute infarction and its extent. Late gadolinium enhancement (LGE) imaging on CMR can accurately distinguish between ischemic and nonischemic patterns of myocardial necrosis and scarring, whereas acute myocardial edema can be detected using T_1_- and T_2_-based mapping techniques.^13^ The latest guidelines from the European Society of Cardiology recommend CMR after ICA in patients with acute myocardial injury and NOCA, because it can determine the cause of the acute myocardial injury and enable appropriate therapy and offer prognostic information.^8^^,^^14^

Given the procedural risks associated with ICA,^14^ early identification of nonischemic causes of suspected NSTEMI is a clinically attractive proposition, because it may obviate the need for ICA in selected cases. In cases of acute NSTEMI associated with multivessel coronary artery disease (CAD), it may help identify the correct IRA and determine downstream myocardial viability (by assessing for transmural LGE with microvascular obstruction and intramyocardial hemorrhage), which may help guide revascularization strategies. Further, CMR can provide important information that may improve the interpretation of ICA findings and the need for adjunctive techniques, such as intracoronary imaging or pressure/flow studies.

Thus, in this prospective study of patients presenting with suspected NSTEMI, we aimed to establish how comprehensive CMR performed before ICA in the acute setting would contribute to the precise diagnosis of the acute myocardial injury, as well as identifying the correct IRA in cases with confirmed acute MI.

## Methods

**Figure 1:**
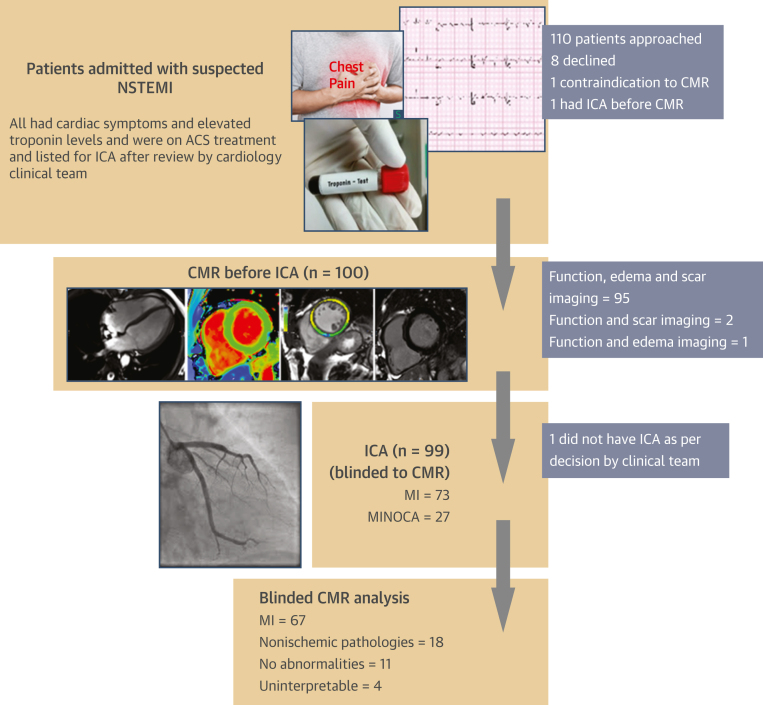
Study Flowchart Patients admitted to hospital with suspected NSTEMI and listed for ICA were prospectively recruited to undergo CMR before ICA, as part of the OxAMI (Oxford Acute Myocardial Infarction) study. The clinicians performing ICA were blinded to CMR findings. CMR experts were also blinded to the ICA findings. ACS = acute coronary syndrome; CMR = cardiac magnetic resonance; ICA = invasive coronary angiogram; MINOCA = myocardial infarction with nonobstructive coronary arteries; NSTEMI = non-ST-segment elevation myocardial infarction.

We prospectively recruited patients admitted with a clinical diagnosis of suspected NSTEMI and scheduled for inpatient ICA at the John Radcliffe Hospital within the Oxford University Hospitals National Health Service Foundation Trust, into the OxAMI (Oxford Acute Myocardial Infarction) study;^15^ the NSTEMI substudy had an active period of recruitment between 2014 to 2015 and 2018 to 2020. Suspected NSTEMI was defined as the presence of new chest discomfort or an equivalent symptom (eg, angina equivalent breathlessness) with an acute rise in high-sensitivity cTnI, with or without acute non–ST-segment elevation ECG changes. Patients recruited into this study had already been reviewed by a member of the clinical cardiology team and were started on dual antiplatelet therapy and anticoagulation, as part of standard clinical care for suspected NSTEMI (Figure 1).^16^ Exclusion criteria to the study included patients with ongoing chest pain, dynamic ECG changes, hemodynamic instability, estimated glomerular filtration rate <30 mL/min/1.73 m^2^, claustrophobia, a contraindication to the MR environment or to gadolinium contrast administration. The study protocol was approved by the Local Ethics Committee (REC:10/H0408/24), and all patients gave written informed consent.

### CMR scanning

All patients in this study underwent CMR before ICA in 3-T MR systems, either MAGNETOM Tim Trio or MAGNETOM Verio (Siemens Healthcare), located at the Oxford Centre for Clinical Magnetic Resonance Research and the Acute Vascular Imaging Centre, respectively, at the John Radcliffe Hospital. CMR protocol included cine steady-state free precession imaging, T_1_ maps (shortened modified Look-Locker inversion recovery [ShMOLLI]),^17^^,^^18^ bright blood T_2_-weighted (T2W) imaging, and early gadolinium enhancement (EGE) and LGE imaging, all offering full left ventricular short-axis coverage from base to apex, as previously described.^19^ LGE images were obtained 10-15 minutes after administration of a total of 0.13 mmol/kg of a gadolinium-based contrast agent (gadoterate meglumine, Dotarem, Guerbet, or gadodiamide, Omniscan, GE Healthcare) in line with OxAMI study protocol.^19^

### CMR image analysis

CMR images were independently analyzed by 3 CMR experts (C.N., A.B., M.S.) using the cvi42 software (Circle Cardiovascular Imaging Inc) and MC-ROI, a dedicated in-house software (programmed in IDL, version 8.7, L3Harris Geospatial). The CMR experts used all available images to make a diagnosis; visual inspection for regional wall motion abnormalities, edema, and myocardial scar was performed on cine, T_1_ maps, and T2W and LGE imaging. Volumetric analysis of biventricular function was performed using short-axis stack of cine imaging. Myocardial T_1_ values higher than the normal range for our scanner (1,124-1,244 milliseconds)^20^ were highlighted using overlay masks in MC-ROI. Quality of T_1_, T_2_, and LGE images were graded on a 4-point scale (good, acceptable, poor, and uninterpretable). CMR analysis was blinded to the ICA findings to derive an independent CMR diagnosis, in line with current CMR guidelines.^13^^,^^21^ In cases of disagreement between the CMR analysts, consensus was reached with input from a fourth CMR expert (V.M.F.). The diagnosis of acute MI required the presence of an ischemic pattern of LGE with associated edema (on T_1_/T_2_ imaging), with or without regional wall motion abnormality (RWMA). Aborted MI was diagnosed in the presence of a RWMA in the distribution of a coronary artery, with or without evidence of acute myocardial edema, but in the absence of LGE. Both acute and aborted MI are referred to as MI hereafter. The determination of the IRA used the American Heart Association 17-segment model of the left ventricle.^22^ Acute myocarditis was diagnosed using the updated Lake-Louise criteria (2018).^13^ Takotsubo cardiomyopathy was diagnosed on the basis of classic RWMAs (eg, apical hypokinesia and ballooning) with associated myocardial edema and in the absence of ischemic LGE. When a CMR scan of interpretable quality showed normal cardiac anatomy, biventricular volumes, systolic global and regional function, valvular motion, T_1_, T_2_, EGE, and LGE, it was reported as having no detectable CMR abnormalities (nonspecific fibrosis at the left and right ventricular insertion points in patients with no CMR other abnormalities were not considered to be abnormal).

### Clinical management

The clinical team members providing care for the patients were blinded to the CMR findings until after the ICA, and they performed investigations and treatment according to current clinical practice. Culprit artery was determined by the interventional cardiologist using available clinical information. MVD was defined as coronary artery lesions with >50% luminal stenosis on ICA in ≥2 coronary arteries. NOCA was diagnosed by the clinical care team when the ICA did not detect obstructive CAD, assessed visually by operator, or by spontaneous coronary artery dissection. If requested by the clinicians, CMR results were provided after the ICA had been performed to aid the clinical management in patients with NOCA.

### Statistical analysis

Normality of data was determined using Kolmogorov-Smirnov test. Normally distributed data are presented as mean ± SD and nonparametric data as median (Q1-Q3). Statistical comparisons were performed using Student’s *t*, Mann-Whitney *U*, chi-square, or Fisher exact test or one-way analysis of variance, as appropriate. Statistical analyses were performed using SPSS (version 25, IBM Corp).

## Results

### Study population: suspected NSTEMI

A total of 100 patients (mean age 62 ± 11 years, 70% were male) were recruited and underwent CMR prior to ICA (Table 1). All patients were admitted with cardiac symptoms (95% with chest pain, with the other 5% having anginal equivalent symptoms) and evidence of acute myocardial injury based on elevated cTnI (median rise of 23 [Q1-Q3: 6-94], fold above the upper limit of normal). Non–ST-segment elevation ECG changes of ischemia were present in 41% of the patients. All patients were clinically managed as suspected NSTEMI, had received dual antiplatelet therapy and anticoagulation, and were scheduled to have an ICA at the time of study recruitment.

**Table 1:** Characteristics for All Patients and Patients Stratified by Diagnosis Made Using CMR Performed Before ICA

	Full Cohort (N = 100)	Findings on CMR Performed Before ICA			*P* Value
		Myocardial Infarction (n = 67)	Nonischemic Pathologies (n = 18)	No Abnormalities (n = 11)	
Demographics					
Age, y	62 ± 12	63 ± 12	63 ± 10	57 ± 11	0.303
Male	70	81	44	64	0.008
BMI, kg/m^2^	29 ± 5	29 ± 5	27 ± 4	27 ± 5	0.189
Hypertension	45	45	50	36	0.774
High cholesterol	26	25	33	27	0.794
Diabetes	12	13	17	0	0.385
Known CAD	15	16	17	9	0.403
CVA	3	5	0	0	0.512
Smoking history	51	55	44	64	0.584
CAD family history	26	16	12	13	0.754
Acute admission					
Chest pain	95	97	82	100	0.046
Ischemic ECG	41	48	24	46	0.218
cTnI rise^a^	31 (7-106)	43 (7-146)	10 (7-34)	5 (2-12)	0.003
SBP, mm Hg	141 ± 25	141 ± 26	136 ± 24	144 ± 20	0.333
Heart rate, beats/min	74 ± 18	74 ± 16	76 ± 25	71 ± 15	0.583
Hemoglobin, g/L	144 (129-150)	140 (131-150)	142 (123-151)	149 (145-151)	0.080
Creatinine, μmol/L	82 ± 18	82 ± 17	82 ± 20	80 ± 17	0.948
ICA findings					
Presumed MI diagnosed based on ICA	73^b^	91	39	36	<0.001
LAD is IRA	31	40	22	0	<0.001
Revascularization	62	75	39	36	0.003
Multivessel CAD	50	54	50	36	0.563
Multivessel revascularization	18	21	17	9	0.429
CMR findings					
Time since symptoms, h	57 (26-70)	47 (25-71)	43 (38-83)	58 (37-68)	0.601
Time since hospitalization, h	33 (20-48)	29 (20-48)	37 (24-48)	37 (22-58)	0.475
Time before ICA, h	4 (2-6)	3 (2-6)	4 (3-6)	7 (2-22)	NS
LVEDVi, mL/m^2^	77 (67-88)	77 (67-87)	78 (65-89)	72 (58-83)	0.296
LVESVi, mL/m^2^	36 (29-41)	36 (29-43)	39 (35-42)	25 (21-32)	0.005
LVEF, %	52 ± 10	51 ± 9	50 ± 12	61 ± 5	0.004
LV mass index, g/m^2^	49 ± 14	53 ± 14	49 ± 13	46 ± 9	0.124
RVEF, %	55 ± 8	55 ± 8	52 ± 13	58 ± 4	0.156

Values are mean ± SD, %, or median (Q1-Q3). *P* values are for comparisons among the 3 subgroups.

BMI = body mass index; CAD = coronary artery disease; CMR = cardiac magnetic resonance; cTnI = cardiac troponin I; CVA = cerebrovascular accident; ECG = electrocardiography; IRA = infarct-related artery; LAD = left anterior descending artery; LVEDVi = left ventricular end-diastolic volume index; LVESVi = left ventricular end-systolic volume index; MI = myocardial infarction; NS = not significant; SBP = systolic blood pressure.

a CTnI rise = fold rise above the upper limit of normal range.

b One patient did not have ICA after CMR but was diagnosed with MI by the clinical team because he had an ICA in the past that had demonstrated significant CAD. Data from the 4 patients with uninterpretable CMR images were not included. CAD refers to any abnormality in the coronary arteries including atherosclerosis and spontaneous coronary artery dissection.

### CMR findings pre-ICA

The CMR scans were performed very early at a median of 33 (Q1-Q3: 20-48) hours after admission to hospital and 4 (Q1-Q3: 2-6) hours before ICA. The complete CMR protocol (cine, T_1_ maps, T2W, EGE, and LGE) was performed on 88% (88 of 100) of patients. Of the remaining 12% (12 of 100) of patients, 7 had cine and LGE imaging alongside either T_1_ maps or T2W imaging, whereas 2 had cine and LGE imaging only, due to inability to tolerate the duration of the scan. The remaining 3 patients experienced claustrophobia and did not have LGE imaging, including 1 patient who did not have any interpretable images available (Figure 1). T_1_, T_2_, and LGE images were of interpretable quality in 97%, 84%, and 87% of the cases, respectively.

**Figure 2:**
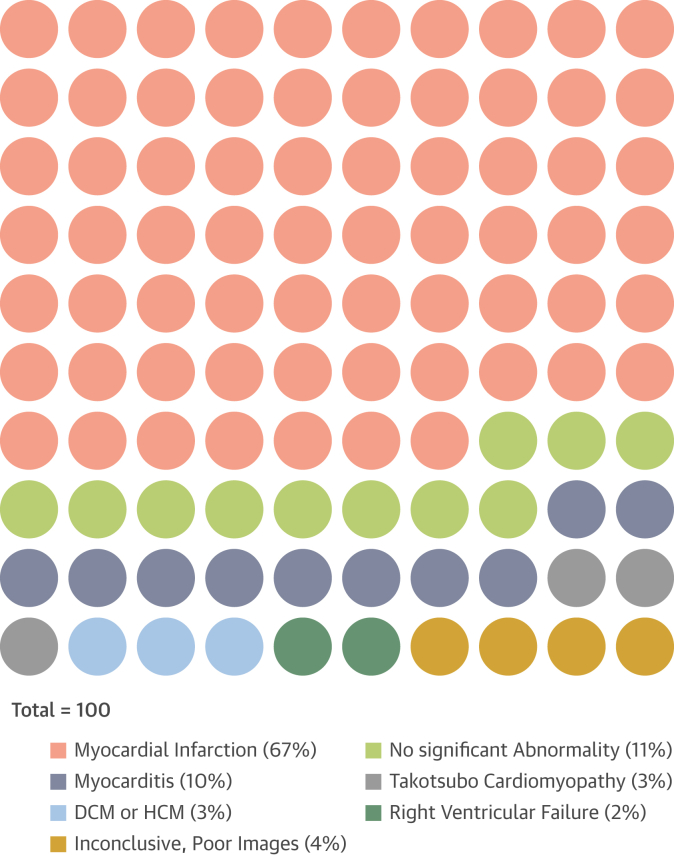
Diagnoses Made Using CMR Prior to ICA Break down (dot-plot) of diagnoses made using CMR performed prior to ICA. DCM = dilated cardiomyopathy; HCM = hypertrophic cardiomyopathy; other abbreviations as in Figure 1.

**Figure 3:**
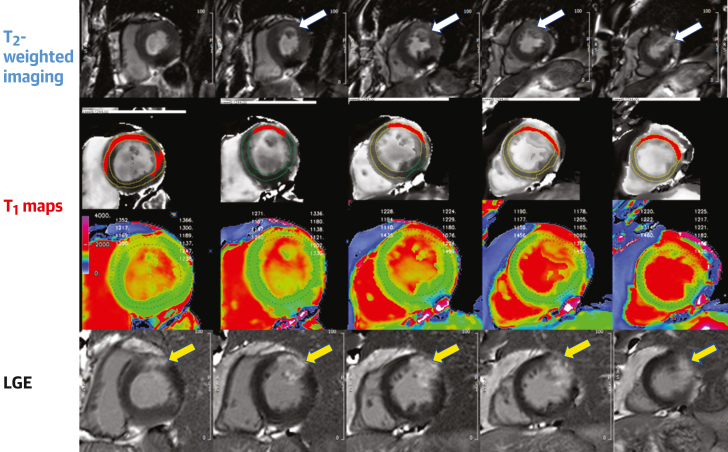
Multiparametric CMR of Acute MI (Top) T_2_-weighted images show hyperenhancement (white arrows) indicating areas of myocardial edema in the anterior and anterolateral walls of the left ventricle. (Middle, first) The overlay masks used to highlight the areas of myocardium with pixels of abnormally high T_1_ values on the shortened modified Look-Locker inversion recovery T_1_ maps (middle, second) (>1,244 milliseconds). (Bottom) Late gadolinium enhancement (LGE) images show hyperenhancement (yellow arrows) indicating areas of myocardial infarction (MI). CMR = cardiac magnetic resonance.

**Figure 4:**
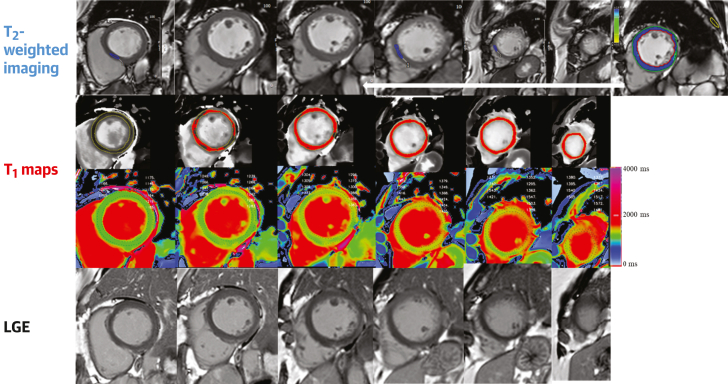
Multiparametric CMR of Takotsubo Syndrome (Top) T_2_-weighted images show global hyperenhancement in midapical left ventricle indicative of intense myocardial edema (the tile in the top-right corresponds to the midventricular slice of the left ventricle with myocardial-skeletal muscle T_2_ signal intensity ratio of >2). (Middle, first) The overlay masks used to highlight the areas of myocardium with pixels of abnormally high T_1_ values on the shortened modified Look-Locker inversion recovery T_1_ maps (middle, second) (>1,244 milliseconds). The edematous segments were hypokinetic on cine imaging. (Bottom) Late gadolinium enhancement (LGE) images show no evidence of significant myocardial fibrosis or infarction. shortened modified Look-Locker inversion recovery

**Figure 5:**
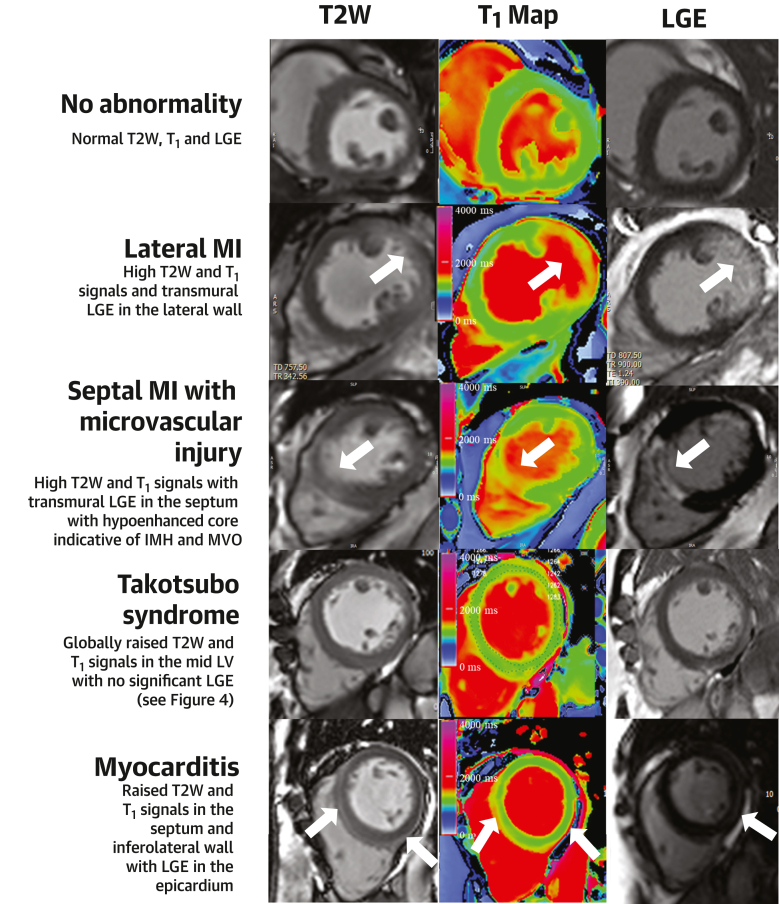
Findings on Multiparametric CMR in Patients With Suspected NSTEMI Multiparametric CMR using T_2_-weighted (T2W) imaging, T_1_ maps, LGE demonstrate normal findings, MI with and without microvascular injury, takotsubo syndrome, and myocarditis. Representative short-axis slices provided for illustration purposes. White block arrows point to areas of focal pathology. IMH = intramyocardial hemorrhage; LV = left ventricle; MVO = microvascular obstruction; other abbreviations as in Figures 1 and 3.

CMR confirmed acute MI in 67% (67 of 100) of patients, of which only 52 were subendocardial infarctions and 15 were transmural MI (7 lateral wall, 5 inferior wall, 2 septum, and 1 anterior wall; likely late-presenting STEMI); nonischemic pathologies in 18% (18 of 100) and no significant abnormalities in 11% (11 of 100), whereas 4% (4 of 100) of the CMR scans was inconclusive due to poor image quality (n = 3) and patient not completing the scan (n = 1) (Figure 2, Figure 3, Figure 4, Figure 5).

The nonischemic diagnoses made on CMR included myocarditis (n = 10), takotsubo cardiomyopathy (n = 3), hypertrophic or dilated cardiomyopathy (n = 3), and isolated right ventricular failure (no right ventricular LGE seen) in 2 patients (Figure 2, Figure 3, Figure 4, Figure 5).

Patients with a CMR diagnosis of confirmed MI (referred to as CMR-MI hereafter) (Figure 3) were predominantly male, when compared to patients with nonischemic etiologies and normal findings (81% vs 33% vs 64%; *P* = 0.008), and had a higher fold troponin rise (median: 43 [Q1-Q3: 7-146] vs 10 [Q1-Q3: 7-34] vs 5 [Q1-Q3: 2-12]; *P* = 0.003). All other baseline clinical characteristics were similar. Left ventricular ejection faction was lower in patients with CMR-MI and nonischemic pathologies, compared to those with no significant CMR abnormalities (51% ± 8% and 50 ± 12% vs 61% ± 5%; *P* = 0.005) (Table 1).

### ICA findings

Based on the ICA features, the clinical care team found a potential culprit coronary artery and made a clinical diagnosis of acute MI in 73% (73 of 100) of the patients (1 did not undergo ICA after CMR, but the clinical care team diagnosed MI based on a previous but recently performed ICA). These patients are referred to as ICA-MI hereafter. Of these, 85% received revascularization (percutaneous coronary intervention [PCI], n = 56; coronary artery bypass graft, n = 6), whereas the remaining 15% received medical therapy alone (n = 11).

MVD was present in 58% (42 of 73) of the patients with ICA-MI. The culprit artery was determined by the interventional cardiologist as the left anterior descending (LAD) in 21 patients, left circumflex in 6 patients, and right coronary artery (RCA) in 9 patients, whereas the culprit vessel was indeterminant in the remaining 6. Multivessel revascularization was performed in 52% (22 of 42) of patients with MVD (PCI, n = 17; coronary artery bypass graft, n = 5). Of the 17 patients who had PCIs to nonculprit arteries, 8 patients had LAD PCI, 4 had left circumflex PCI, 4 had RCA PCI, and 1 patient had PCI to 2 nonculprit arteries.

At the time of ICA, NOCA was found in 27% (27 of 100) of patients. These patients are referred to as ICA-NOCA hereafter and were presumed to have sustained a MINOCA (Table 2).

**Table 2:** Comparison of Diagnoses Made on ICA and CMR

		CMR Diagnosis (Pre-ICA)			Inconclusive (Incomplete Scan or Poor Image Quality)	Total
		MI	Nonischemic Causes	No Significant Abnormalities		
Clinical diagnosis based on ICA	Obstructive CAD (presumed MI)	61	7	4	1	73
	NOCA (presumed MINOCA)	6	11	7	3	27
Total		67 (transmural MI, n = 15; subendocardial MI, n = 52)	18	11	4	100

Values are n. CMR provided a diagnosis in 85 patients and led to a reclassification to a non-MI diagnosis in 29 patients. Of the 67 patients with MI, 52 were subendocardial infarctions, and 15 were transmural infarctions (likely a late-presenting STEMI instead of an acute NSTEMI). CMR did not find any abnormalities in 11 patients, of which 4 were found to have obstructive coronary artery disease and were treated as having had an acute coronary syndrome. ICA-NOCA nonischemic cases breakdown: 6 myocarditis, 2 takotsubo, 3 cardiomyopathies. ICA-obstructive CAD nonischemic causes breakdown: 4 myocarditis, 1 takotsubo, 2 RV failure.

MINOCA = myocardial infarction with nonobstructive coronary arteries; NOCA = nonobstructive coronary arteries; NSTEMI = non–ST-segment elevation myocardial infarction; other abbreviations as in Table 1.

At discharge, using routine non-CMR clinical diagnostics (history, ECG, echocardiogram, and ICA), 5 of 27 were diagnosed with takotsubo syndrome, whereas 1 each were diagnosed as heart failure and hypertrophic cardiomyopathy. The remainder (n = 20) continued to receive a presumed diagnosis of MINOCA and were discharged on dual antiplatelet therapy. They had a smaller troponin rise when compared to those with ICA-MI (9 [Q1-Q3: 6-24] vs 43 [Q1-Q3: 8-140] fold; *P* = 0.019).

### Comparison of diagnoses made on CMR and ICA

Overall, the presumed working diagnosis of NSTEMI in the 100 patients was only concordant with CMR-confirmed MI in 67% (67 of 100) patients (61 patients in the ICA-MI group, and 6 patients in the ICA-NOCA group) (Table 2). Of note, 15 were transmural infarctions (7 lateral wall, 5 inferior wall, 2 septum, and 1 anterior wall) likely to be late-presenting STEMIs rather than acute NSTEMIs).

**Figure 6:**
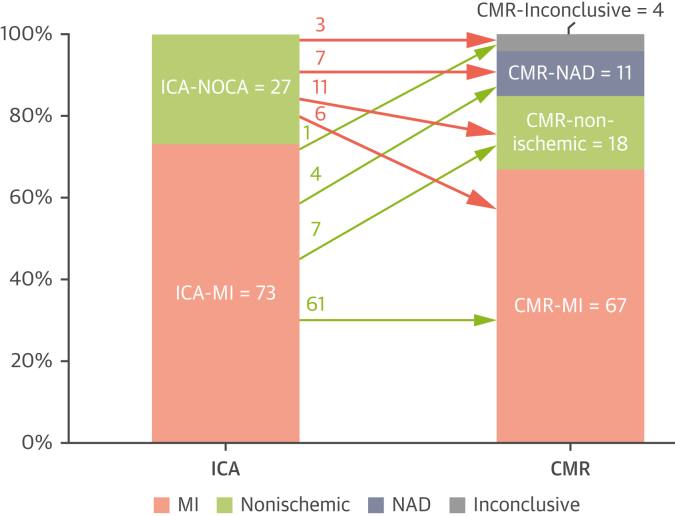
Reclassification of ICA Diagnoses by CMR Reclassification of diagnoses by CMR in patients treated for MI on ICA (n = 73) and nonobstructed coronary arteries (NOCA) on ICA (n = 27). Nonischemic causes include myocarditis, dilated cardiomyopathy, hypertrophic cardiomyopathy, takotsubo cardiomyopathy, and right ventricular failure. NAD = no significant abnormality detected; other abbreviations as in Figures 1 and 3.

In the patients with a presumed diagnosis of MI based on angiographic findings (ICA-MI; n = 73), CMR confirmed the presence of MI in only 84% (61 of 73); in the remaining 16% (12 of 73) of patients with ICA-MI, CMR demonstrated nonischemic causes in 7 of 12 (58%), did not detect any MI or other abnormality in 4 of 12 (33%) despite good image quality, and was inconclusive in 1 case due to poor image quality (Central Illustration, Figure 6). In patients who had normal findings on CMR pre-ICA (11%, 11 of 100), the majority (64%, 7 of 11) had NOCA on ICA, but 36% (4 of 11) of patients were found to have obstructive CAD on ICA.

**Central Illustration:**
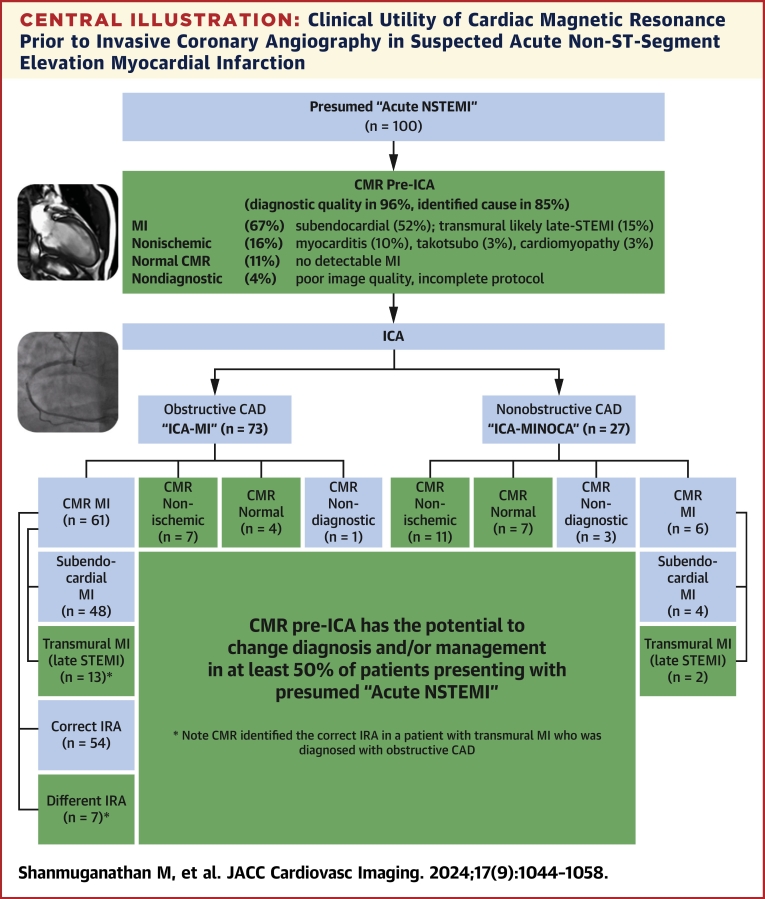
Clinical Utility of Cardiac Magnetic Resonance Prior to Invasive Coronary Angiography in Suspected Acute Non-ST-Segment Elevation Myocardial Infarction Cardiac magnetic resonance (CMR) prior to invasive coronary angiography (ICA) produced a diagnosis in 85% and has the potential to change diagnosis and/or clinical management in at least 50% of the patients. Note: CMR was not interpretable in 4% of patients due to incomplete scan or poor image quality. CAD = coronary artery disease; IRA = infarct related artery; MINOCA = myocardial infarction with nonobstructive coronary arteries; NSTEMI = non–ST-segment elevation myocardial infarction.

### CMR diagnoses of patients with ICA-NOCA

In patients with ICA-NOCA (n = 27), CMR diagnosed nonischemic pathologies in 41% (11 of 27: 6 myocarditis, 3 cardiomyopathy, 2 takotsubo), MI (true MINOCA) in 22% (6 of /27), and no significant abnormalities in 26% (7 of 27). The remaining 3 scans were nondiagnostic due to incomplete scan or poor image quality (Central Illustration, Figure 6).

The 3 subgroups within the ICA-NOCA cohort who had a CMR diagnosis (nonischemic, true MINOCA, and normal) were of similar age (65 ± 10 years vs 65 ± 7 years vs 56 ± 12 years; *P* = 0.174), and had similar burden of chest pain at presentation (85% vs 83% vs 100%; *P* = 0.248), ischemic ECG abnormalities (*P* = 0.135) and level of troponin (*P* = 0.208).

### Identification of the IRA on ICA vs CMR

Among patients diagnosed with MI on CMR and obstructive CAD on ICA (n = 61), there was diagnostic concordance in only 54 patients (89%). CMR identified a different IRA in 7 patients (11%), and all 7 patients had MVD, of whom 3 patients had PCI; among these, the IRA mismatches between ICA and CMR were RCA-LAD, RCA-LAD, and LAD-RCA. Among the 4 who did not have PCI, the IRA was not determined on ICA, but was inferred on CMR as LAD in 3 and RCA in 1. Two of these patients had coronary artery bypass graft and the other 2 were medically managed.

Of 42 patients with ICA-MI and MVD, 35 patients (83%) had MI on CMR, 3 had nonischemic findings, and 3 had normal findings, whereas 1 patient’s CMR was inconclusive.

In our center, the clinical team did not routinely perform clinical transthoracic echocardiogram (TTE) in all the patients before diagnosing NSTEMI and listing them for an ICA. TTE was performed before ICA in 32% of the cohort (32 of 100), of which 18 showed RWMA in keeping with NSTEMI, 13 showed normal findings, and 1 showed mild global hypokinesia of the left ventricle. A direct comparison of diagnoses in this small group (n = 32) showed that CMR agreed with the TTE diagnosis in only one-half of patients (n = 16); (NSTEMI, n = 14; normal, n = 2).

### Potential diagnostic and clinical utility of CMR pre-ICA

Overall, in patients presenting with suspected acute NSTEMI, CMR pre-ICA confirmed MI in 67% of patients, and had the potential to change diagnosis and/or management in 50% of patients (Central Illustration) by: 1) reclassifying the presumed diagnosis of acute NSTEMI into a) probable late-presentation transmural MI in 15% (15 of 100) of patients, or b) nonischemic pathologies (no MI) in 18% (18 of 100) of patients; 2) identifying a different IRA in patients diagnosed with both MI on CMR and obstructive CAD on ICA in 11% (7 of 61) of patients (1 had a transmural MI); or 3) providing a diagnosis of no MI with normal CMR findings in 11% (11 of 100) of patients (who would still need imaging of the coronary arteries). Note that CMR helped identify the correct IRA in 1 patient with transmural MI.

## Discussion

To the best of our knowledge, this is the largest prospectively designed study to-date to investigate the diagnostic utility of a comprehensive CMR (including parametric mapping and full heart coverage) pre-ICA in patients with suspected acute NSTEMI. This study found the following:

1. An acute multiparametric CMR protocol (to assess ventricular function, edema, and scar) can be successfully completed in 96% of stable patients presenting with acute NSTEMI who were stable, scheduled for an ICA, and deemed suitable to undergo the research protocol.
2. A pre-ICA CMR confirmed a subendocardial MI in only 52%, whereas alternate diagnoses were transmural MI (likely late-presenting STEMI) in 15%, nonischemic pathology in 18%, and normal in 11%.
3. If only relying on the ICA to diagnose MI based on findings of obstructive CAD (n = 73), CMR confirmed MI in only 84%, but showed no MI in 15% of patients (nonischemic pathologies in 10% and no significant abnormalities in 5%) and was inconclusive in 1%.
4. In patients with NOCA on ICA (n = 27), CMR detected nonischemic etiologies in 41% (n = 11) and true MINOCA in only 22% (n = 6). CMR showed no significant abnormalities in 26% (n = 7) of these patients, who can be offered the reassurance of having a normal ICA and CMR.
5. In patients who had normal findings on CMR pre-ICA (11%, 11 of 100), the majority (64%, 7 of 11) had NOCA on ICA, but 36% (4 of 11) of patients were found to have obstructive CAD on ICA.
6. A CMR-first strategy has the potential to change diagnosis and/or management in at least 50% of patients presenting with suspected acute NSTEMI, by reclassifying the presumed diagnosis of subendocardial MI into transmural MI (15%∗), nonischemic pathology (18%) and no MI (11%), and identifying a new IRA in patients with obstructive CAD on ICA and CMR-confirmed MI (7%∗), to guide revascularization and clinical decision-making.

In current clinical practice, a working diagnosis of MINOCA is made when a patient with acute myocardial injury is found to have NOCA on ICA.^10^ However, not all in this group have sustained an MI, and thus intracoronary imaging and vasoreactivity testing and/or CMR are recommended in all cases of NOCA to confidently exclude MI due to coronary pathologies, such as acute rupture of a small atherosclerotic plaque, coronary artery dissection, vasospasm, or embolism.^10^^,^^14^^,^^23^

However, the role of acute CMR imaging as a gatekeeper to ICA in patients with acute myocardial injury and suspected NSTEMI is less well-established, which has led researchers to investigate this in 3 previous studies.^24^, ^25^, ^26^ One of these studies relied only on cine imaging (for wall motion and ventricular function) and LGE imaging (for scar assessment),^25^ whereas the other 2 had also employed T2W imaging (for imaging myocardial edema) and stress perfusion imaging (for detection of ischemia).^24^^,^^26^ None of them had employed parametric mapping, as we have done in our study, which are considered to be more sensitive in detecting both ischemic and nonischemic myocardial injury, compared to more conventional MR imaging methods.^13^^,^^27^, ^28^, ^29^, ^30^ In their observational study of 114 patients with suspected NSTEMI, Heitner et al,^25^ focused on using CMR to identify the IRA in selected patients presenting with their first MI, but excluded patients with a potential nonischemic cause for their presentation. Their CMR protocol (only cine and LGE) produced a diagnosis in 86% (including a nonischemic diagnosis in 15% of patients), and the CMR led to a new IRA diagnosis in 31% of patients with NSTEMI.^25^ Smulders et al^24^ randomized patients with suspected NSTEMI to either CMR (n = 60) or computed tomography coronary angiogram (n = 70) or ICA (n = 68) in the CARMENTA (CARdiovascular Magnetic rEsoNance imaging and computed Tomography Angiography) trial, and found that a CMR-first strategy obviated the need for ICA in 13% of patients, and that the clinical outcomes of the 3 cohorts were similar at 1 year. Among those who underwent CMR (n = 60; cine, T2W, LGE, and stress perfusion imaging), the diagnostic yield was 79% (MI in 77%, myocarditis in 2%) with equivocal and normal findings in 7% and 13%, respectively. NSTEMI.^25^ Van Cauteren et al^26^ undertook a subanalysis of the CARMENTA trial in 51 patients, who had both CMR and ICA, which yielded a diagnosis only in 76% in the patients (normal findings in 24%); the protocol consisted of cine, T2W imaging, and LGE in all patients and stress perfusion imaging in 19 patients with normal/equivocal findings on the initial 3 CMR sequences).^26^ It should be noted that the diagnostic yield of studies examining patients presenting with suspected acute NSTEMI depend heavily on patient selection, the timing of CMR after the acute presentation, and comprehensiveness of CMR imaging protocols.

Our diagnostic yield of 85% with CMR-first strategy using cine, T_1_ maps, and T2W and LGE imaging, all offering whole-heart coverage, is higher than that reported in the studies by Van Cauteren et al^26^ (76%) and Smulders et al^24^ (79%) (both of whom used stress perfusion imaging in addition to cine, T2W, and LGE). Heitner et al^25^ used cine and LGE only to focus on diagnosing the IRA in NSTEMI and had a different patient selection to our study (excluded patients clinically thought to have a nonischemic presentation). Heitner et al^25^ also used conventional cTnI assays to diagnose acute myocardial injury, whereas our center’s use of high-sensitivity cTnI assays most likely resulted in the recruitment of some patients with smaller magnitude of acute myocardial injury. This highlights the incremental diagnostic value provided by whole-heart T_1_- and T_2_-based edema imaging in the detection of subtle acute myocardial injury and myocarditis.

In our study, the patients who had normal CMR findings pre-ICA had much lower cTnI rise than the cohorts with ischemic and nonischemic diagnoses (5-fold vs 43-fold and 10-fold, respectively; *P* = 0.004) (Table 1). Of the 11 patients with normal CMR findings pre-ICA, 7 (64%) also had NOCA on ICA; therefore, it is possible that their acute myocardial injury was beyond the resolution of CMR imaging. However, the remaining 4 patients were found to have obstructive CAD on ICA; the lack of evidence of RWMA or myocardial edema or infarction in these 4 patients suggest that their presentation may have been due to mild myocardial ischemia leading to mild elevation of high-sensitivity cTnI levels, which may not be within the sensitivity of multiparametric CMR. CMR may not be sensitive enough to detect mild injury to the thin right ventricular myocardium or very small myocardial infarctions of either ventricles associated with small CTnI rises, as previously described.^31^ Therefore, whereas a patient with no detectable CMR abnormalities has a high chance of NOCA (64% in our study), an assessment of their coronary arteries should still be performed to exclude obstructive CAD. The detection of atherosclerotic CAD may warrant treatment that may otherwise not be initiated if only CMR were undertaken in this setting.

On the other hand, in our study, CMR demonstrated that a significant proportion of patients with CMR-MI pre-ICA (22%; 15 of 67) had a transmural acute MI. These were either in the lateral (posterior) wall or inferior wall, which may be electrically silent on ECG, due to lower sensitivity of the ECG in detecting acute MIs in these territories in some patients. These patients may have therefore suffered from a complete occlusion of a coronary artery prior to their presentation to hospital when their ECGs did not demonstrate a clear ST-segment elevation or were evolving. Knowing the location and the extent of MI before the ICA in such patients has the potential to inform the clinicians of the need and benefit of revascularization.

We believe that a CMR-first strategy is both safe and clinically feasible, as shown in our study and those by other groups.^24^^,^^25^ When used as a gatekeeper to ICA in acute myocardial injury, it has the potential to save health care costs, because ICA may be associated with a long hospital stay and procedural complications. Our findings should inform large pragmatic multicenter randomized control trials of a CMR-first strategy vs ICA-first strategy vs a hybrid (CMR and ICA) strategy, in determining the causes of acute myocardial injury in patients with suspected NSTEMI. The recent advances in contrast-free CMR for tissue characterization^32^^,^^33^ and coronary imaging (coronary CMR angiogram)^34^ may further this approach with the claimed 3-fold faster and cheaper imaging solutions and may help CMR to become a first-line noninvasive test for patients with acute myocardial injury. Other options to replace ICA as a first-line diagnostic test include a hybrid noninvasive imaging strategy using high-resolution computed tomography cardiac angiogram (CTCA) and CMR in all comers before proceeding to ICA; this approach may offer incremental diagnostical power in cases where either test fails to detect the cause of acute myocardial injury. Even when CMR suggests evidence of a nonischemic pathology, further investigation for CAD (eg, coronary computed tomography angiography) may be required. Ultimately, the decision to pursue further testing can be left at the clinician's discretion based on the clinical characteristics and suspicion. Any of these strategies will need to be tested for clinical effectiveness and cost-effectiveness in large trials and will require the availability of scanners, fast imaging protocols, and prompt reporting of scans to aid wider adoption in the acute setting.

### Study limitations

Comprehensive evaluation with intracoronary imaging was not performed in most of the cases, which could have improved ICA’s performance in diagnosing acute plaque rupture/other acute coronary pathology and true NOCA alike. Our definition of match between ICA and CMR in identifying the IRA was limited to the 3 main coronary arteries and is based on the standard American Heart Association 17-segment model, assuming normal coronary anatomy. Our scanner did not have a fully validated T_2_ mapping sequence at the time of implementation over a decade ago, which is now an integral part of contemporary multiparametric CMR imaging to detect myocardial edema; however, we did use both a well-validated T_1_ mapping sequence for detecting acute edema^27^ and T2W imaging, which are well-established methods in detecting edema and as previous published.^35^, ^36^, ^37^, ^38^ Our study recruited only the patients deemed suitable for ICA (and only during weekday hours when research staff were available) and did not include those medically managed for suspected NSTEMI. On a related point, the relatively high proportion of patients with NOCA (27%) in our cohort may have been due to the exclusion of patients with high-risk features of CAD (ischemia and hemodynamic instability).

## Conclusions

In patients presenting with suspected NSTEMI, a CMR-first strategy identified MI in 67%, nonischemic pathologies in 18%, and normal findings in 11%. Accordingly, CMR has the potential to affect at least 50% of all patients by reclassifying their diagnosis or altering their potential management.

Perspectives **COMPETENCY IN MEDICAL KNOWLEDGE:** Most patients hospitalized with suspected NSTEMI undergo ICA. However, a significant proportion of patients are found to have NOCA and are subsequently diagnosed with a nonischemic etiology (often with the use of CMR). Moreover, a larger proportion of patients have multivessel coronary artery disease, rendering the identification of the IRA tricky. In this observational study, CMR was performed prior to ICA in stable patients hospitalized with suspected NSTEMI and listed for ICA. CMR showed myocardial infarction in 67%, nonischemic pathologies in 18% and normal findings in 11% and demonstrated the potential to change clinical management in approximately one-half of the patients.

**TRANSLATIONAL OUTLOOK:** Large randomized clinical trials with long-term follow-up are needed to assess the clinical and cost-effectiveness of a noninvasive imaging strategy (followed by ICA if needed) in patients presenting with suspected NSTEMI.

## Funding Support and Author Disclosures

The OxAMI study is supported by a British Heart Foundation (BHF) Centre of Research Excellence (CRE) Oxford (RE/13/1/30181), and the National Institute for Health Research Oxford Biomedical Research Centre. Dr Shanmuganathan has received funding from the Alison Brading Memorial Graduate Scholarship in Medical Science, Lady Margaret Hall, University of Oxford. Dr Burrage has received support from a British Heart Foundation Clinical Research Training Fellowship (FS/19/65/34692). Dr Gara has received a European Society of Cardiology, EACVI Research grant. Dr Piechnik has received support from the BHF CRE Oxford (RE/18/3/34214); and has patent authorship rights for U.S. patent 9285446 B2 (systems and methods for Shortened Look Locker Inversion Recovery [Sh-MOLLI] cardiac gated mapping of T1), granted March 15, 2016; intellectual properties are owned and managed by Oxford University Innovations. Dr Channon has received funding from a BHF Chair award (CH/16/1/32013). Dr Ferreira has received funding from the BHF, BHF CRE Oxford, and National Institute for Health Research Oxford Biomedical Research Center. The funders were not involved in the design and conduct of the study, in the collection, analysis, and interpretation of the data, and in the preparation, review, or approval of the manuscript. All other authors have reported that they have no relationships relevant to the contents of this paper to disclose.

## Footnotes

∗ CMR helped identify the correct IRA in a patient with transmural MI.

## References

[1] Amsterdam E.A., Wenger N.K., Brindis R.G. (2014). 2014 AHA/ACC guideline for the management of patients with non–ST-elevation acute coronary syndromes: a report of the American College of Cardiology/American Heart Association Task Force on Practice Guidelines. J Am Coll Cardiol.

[2] Bueno H., James S., Camm A.J., Lüscher T.F., Maurer G., Serruys P.W. (2018). The ESC Textbook of Cardiovascular Medicine.

[3] NICOR Myocardial Ischaemia/MINAP (Heart Attack audit). https://www.nicor.org.uk/national-cardiac-audit-programme/heart-attack-audit-minap.

[4] Tamis-Holland J.E., Jneid H. (2018). Myocardial infarction with nonobstructive coronary arteries (MINOCA): it’s time to face reality. J Am Heart Assoc.

[5] Dastidar A.G., Baritussio A., De Garate E. (2019). Prognostic role of CMR and conventional risk factors in myocardial infarction with nonobstructed coronary arteries. JACC Cardiovasc Imaging.

[6] Sörensson P., Ekenbäck C., Lundin M. (2021). Early comprehensive cardiovascular magnetic resonance imaging in patients with myocardial infarction with nonobstructive coronary arteries. JACC Cardiovasc Imaging.

[7] Liang K., Nakou E., Del Buono M.G., Montone R.A., D’Amario D., Bucciarelli-Ducci C. (2022). The role of cardiac magnetic resonance in myocardial infarction and non-obstructive coronary arteries. Front Cardiovasc Med.

[8] Mileva N., Paolisso P., Gallinoro E. (2023). Diagnostic and prognostic role of cardiac magnetic resonance in MINOCA. JACC Cardiovasc Imaging.

[9] Niccoli G., Scalone G., Crea F. (2015). Acute myocardial infarction with no obstructive coronary atherosclerosis: mechanisms and management. Eur Heart J.

[10] Singh T., Chapman A.R., Dweck M.R., Mills N.L., Newby D.E. (2021). MINOCA: a heterogenous group of conditions associated with myocardial damage. Heart.

[11] Ibrahim H., Sharma P.K., Cohen D.J. (2017). Multivessel versus culprit vessel–only percutaneous coronary intervention among patients with acute myocardial infarction: insights from the TRANSLATE-ACS observational study. J Am Heart Assoc.

[12] Treibel T.A., White S.K., Moon J.C. (2014). Myocardial tissue characterization: histological and pathophysiological correlation. Curr Cardiovasc Imaging Rep.

[13] Ferreira V.M., Schulz-Menger J., Holmvang G. (2018). Cardiovascular magnetic resonance in nonischemic myocardial inflammation: expert recommendations. J Am Coll Cardiol.

[14] Collet J.-P., Thiele H., Barbato E. (2021). 2020 ESC guidelines for the management of acute coronary syndromes in patients presenting without persistent ST-segment elevation: the Task Force for the management of acute coronary syndromes in patients presenting without persistent ST-segment elevation of the European Society of Cardiology (ESC). Eur Heart J.

[15] Scarsini R., Shanmuganathan M., De Maria G.L. (2021). Coronary microvascular dysfunction assessed by pressure wire and CMR after STEMI predicts long-term outcomes. JACC Cardiovasc Imaging.

[16] NHS Oxford University Hospitals NHS Foundation Trust Clinical biochemistry. Troponin I. https://www.ouh.nhs.uk/biochemistry/tests/tests-catalogue/troponin-1.aspx.

[17] Piechnik S.K., Ferreira V.M., Dall’Armellina E. (2010). Shortened modified Look-Locker inversion recovery (ShMOLLI) for clinical myocardial T1-mapping at 1.5 and 3 T within a 9 heartbeat breathhold. J Cardiovasc Magn Reson.

[18] Zhang Q., Werys K., Popescu I.A. (2021). Quality assurance of quantitative cardiac T1-mapping in multicenter clinical trials: a T1 phantom program from the Hypertrophic Cardiomyopathy Registry (HCMR) study. Int J Cardiol.

[19] Shanmuganathan M., Masi A., Burrage M.K. (2023). Acute response in the noninfarcted myocardium predicts long-term major adverse cardiac events after STEMI. JACC Cardiovasc Imaging.

[20] Shanmuganathan M., Kotronias R.A., Burrage M.K. (2023). Acute changes in myocardial tissue characteristics during hospitalization in patients with COVID-19. Front Cardiovasc Med.

[21] Schulz-Menger J., Bluemke D.A., Bremerich J. (2020). Standardized image interpretation and post-processing in cardiovascular magnetic resonance—2020 update. J Cardiovasc Magn Reson.

[22] Cerqueira M.D., Weissman N.J., Dilsizian V. (2002). American Heart Association Writing Group on Myocardial Segmentation and Registration for Cardiac Imaging. Standardized myocardial segmentation and nomenclature for tomographic imaging of the heart: a statement for healthcare professionals from the Cardiac Imaging Committee of the Council on Clinical Cardiology of the American Heart Association. Circulation.

[23] Gudenkauf B., Hays A.G., Tamis-Holland J. (2022). Role of multimodality imaging in the assessment of myocardial infarction with nonobstructive coronary arteries: beyond conventional coronary angiography. J Am Heart Assoc.

[24] Smulders M.W., Kietselaer B.L.J.H., Wildberger J.E. (2019). Initial imaging-guided strategy versus routine care in patients with non–ST-segment elevation myocardial infarction. J Am Coll Cardiol.

[25] Heitner J.F., Senthilkumar A., Harrison J.K. (2019). Identifying the infarct-related artery in patients with non–ST-segment–elevation myocardial infarction. Circ Cardiovasc Interv.

[26] van Cauteren Y.J.M., Smulders M.W., Theunissen R.A.L.J. (2021). Cardiovascular magnetic resonance accurately detects obstructive coronary artery disease in suspected non-ST elevation myocardial infarction: a sub-analysis of the CARMENTA trial. J Cardiovasc Magn Reson.

[27] Ferreira V.M., Piechnik S.K., Dall’Armellina E. (2012). Non-contrast T1-mapping detects acute myocardial edema with high diagnostic accuracy: a comparison to T2-weighted cardiovascular magnetic resonance. J Cardiovasc Magn Reson.

[28] Ferreira V.M., Piechnik S.K., Dall’Armellina E. (2013). T(1) mapping for the diagnosis of acute myocarditis using CMR: comparison to T2-weighted and late gadolinium enhanced imaging. JACC Cardiovasc Imaging.

[29] Dall’Armellina E., Karia N., Lindsay A.C. (2011). Dynamic changes of edema and late gadolinium enhancement after acute myocardial infarction and their relationship to functional recovery and salvage index. Circ Cardiovasc Imaging.

[30] Ferreira V.M., Piechnik S.K., Dall’Armellina E. (2014). Native T1-mapping detects the location, extent and patterns of acute myocarditis without the need for gadolinium contrast agents. J Cardiovasc Magn Reson.

[31] Williams M.G.L., Liang K., De Garate E. (2022). Peak troponin and CMR to guide management in suspected ACS and nonobstructive coronary arteries. JACC Cardiovasc Imaging.

[32] Zhang Q., Burrage M.K., Shanmuganathan M. (2022). Artificial intelligence for contrast-free MRI: scar assessment in myocardial infarction using deep learning-based virtual native enhancement. Circulation.

[33] Zhang Q., Burrage M.K., Lukaschuk E. (2021). Toward replacing late gadolinium enhancement with artificial intelligence virtual native enhancement for gadolinium-free cardiovascular magnetic resonance tissue characterization in hypertrophic cardiomyopathy. Circulation.

[34] Nazir M.S., Bustin A., Hajhosseiny R. (2022). High-resolution non-contrast free-breathing coronary cardiovascular magnetic resonance angiography for detection of coronary artery disease: validation against invasive coronary angiography. J Cardiovasc Magn Reson.

[35] Liu D., Borlotti A., Viliani D. (2017). CMR native T1 mapping allows differentiation of reversible versus irreversible myocardial damage in ST-segment-elevation myocardial infarction: an OxAMI study (Oxford Acute Myocardial Infarction). Circ Cardiovasc Imaging.

[36] Alkhalil M., Borlotti A., De Maria G.L. (2018). Dynamic changes in injured myocardium, very early after acute myocardial infarction, quantified using T1 mapping cardiovascular magnetic resonance. J Cardiovasc Magn Reson.

[37] Dall’Armellina E., Piechnik S.K., Ferreira V.M. (2012). Cardiovascular magnetic resonance by non contrast T1-mapping allows assessment of severity of injury in acute myocardial infarction. J Cardiovasc Magn Reson.

[38] Cuculi F., Dall’Armellina E., Manlhiot C. (2014). Early change in invasive measures of microvascular function can predict myocardial recovery following PCI for ST-elevation myocardial infarction. Eur Heart J.

